# Effects of the dietary digestible fiber-to-starch ratio on pellet quality, growth and cecal microbiota of Angora rabbits

**DOI:** 10.5713/ajas.19.0221

**Published:** 2019-08-03

**Authors:** Guiqin Yang, Fei Zhao, He Tian, Jiantao Li, Dongxin Guo

**Affiliations:** 1 College of Animal Husbandry and Veterinary Medicine, Shenyang Agricultural University, Shenyang, Liaoning 110866, China

**Keywords:** Digestible Fiber, Starch, Pellet Quality, Cecal Microbiota, Angora Rabbit

## Abstract

**Objective:**

Substituting starch with digestible fiber (dF) can improve digestive health of rabbits and reduce costs. Therefore, it is necessary to develop a criterion for dF and starch supply. Effects of the dietary dF-to-starch ratio on pellet quality, growth and cecal microbiota of Angora rabbits were evaluated.

**Methods:**

Five isoenergetic and isoproteic diets with increasing dF/starch ratios (0.59, 0.66, 0.71, 1.05, and 1.44) were formulated. A total of 120 Angora rabbits with an average live weight of 2.19 kg were randomly divided into five groups with four replicates. At the end of 40 day feeding trial, cecal digesta were collected to analyse microbiota.

**Results:**

The results showed that the dF/starch ratio had linear effects on pellet variables (p< 0.01). When the dF/starch ratio was 1.44, the pellets had the lowest powder and highest durability. The dF/starch ratio had unfavorable linear effects on growth variables (p<0.001). When analyzed by quadratic regression, the optimal dF/starch ratios for average weight gain and feed/gain were 0.59 and 0.74, respectively. There were differences in wool yield, fiber length and fiber diameter caused by the dF/starch ratio (p<0.05), and the dF/starch ratios that ranged from 0.66 to 1.06 were appropriate for good results. The cecal microbiota operational taxonomic unit (OTU) number index in the 1.05 dF/starch treatment was higher than that in the 0.66 and 0.71 dF/starch treatments. The higher dF/starch ratio resulted in a higher cecal microbiota OTU number index (p<0.05). The proportion of *Ruminococcus* in the 0.71 dF/starch treatment was higher than that in the 0.59 dF/starch treatment (p<0.05)

**Conclusion:**

The most suitable dF/starch ratio for feed pellet quality is 1.44, and for rabbit growth the optimal range of ratios is from 0.59 to 0.74. With combination of the wool growth, output cost, and cecal microbiota, we suggest that a dietary dF/starch ratio ranging from 0.74 to 1.06 is optimal.

## INTRODUCTION

Angora rabbit produces 1.0 to 1.4 kg/yr of pure fine animal fiber. This represents about 30% of its live weight. However, there is a considerable paucity of information on Angora rabbit nutrition compared with published work on the production of meat from rabbits or wool from sheep. The special feed for Angora rabbit is also extremely rare on the market. Therefore, the nutritional research of Angora rabbit requires particular attention.

The carbohydrate fraction (fiber and starch) is a major component of commercial rabbit diets. Fiber and starch both affect the stability of the microbial ecosystem and gut health. Dietary fiber accounts for 40% to 50% of the total diet. Its importance is related to the influence on feed consumption, rate of digestive passage and role as a substrate for cecal microbiota [1]. Sufficient dietary fiber is essential to prevent digestive problems during rabbit growth. Moreover, dietary fiber also influences pelleting capacity of the feed [2]. The role of dietary fiber and starch has been studied and a protective effect of lignocellulose (poorly digested fibre = acid detergent fiber [ADF]) on gut health has been demonstrated. Favorable effects of digestible fiber (dF = hemicelluloses+ pectin) are also recognized [3]. By-products of crop production, such as brans rich in dF, are commonly used to produce rabbit feed. Hemicelluloses and pectin are the main forms of polysaccharide in rabbit feed. In the rabbits, the dietary dF becomes an energy source derived from microbiota activity that takes place mainly in the large intestine – cecum and proximal colon. When a sufficient supply of ADF (at least 18%) is provided, the replacement of some starch by dF fractions is advised [4,5]. The effects of replacement of dF by starch and *vice versa* on digestive parameters was described by Gidenne and Perez [6], and Gidenne and Bellier [7]. Substitution of protein by dF also led to a significant improvement in the digestive health status of growing rabbits, without significant impairment of growth [5,8]. It has been recommended that dF/ADF ratio remain under 1.3 for diets having an ADF level >15% [9]. Because dF only represents a fraction of the total dietary carbohydrate, this recommendation can be met with diets containing different concentrations of starch due to the varying levels of fiber and simultaneously an inverse variation in the level of starch (since rabbits are fed with complete diets) [10]. Therefore, it is necessary to develop a criterion for dF and starch supply. The substitution of starch for fiber has been studied for rabbit doe diets [11]. However, few studies have examined the impact of dF and (or) starch on the production traits of the Angora rabbit, especially on pellet quality. We hypothesized that feeding a moderate increase of the dietary dF-to-starch (dF/starch) ratio would enhance pellet quality, and maintain normal growth of Angora rabbits. This study aimed to evaluate if the dF/starch ratio of the diet would affect the pellet quality, growth and cecal microbiota of Angora rabbits.

## MATERIALS AND METHODS

### Animals and housing

The experiment was conducted in accordance with the Institutional Animal Care and Use Committee at Shenyang Agricultural University (Shenyang, China). It was carried out at the Jinhua Angora Rabbit Industrial Park (Meihekou, China), during the period from October 2016 to December 2017. A total of 120 growing female (since males are not frequently employed because of their lower wool production, about 5% to 10% less) Wan strain Angora rabbits (83 d old and 2.19±0.07 kg) were selected and randomly allocated in galvanized metal wire cages with automatic drinkers with nipples, to five treatments each with 4 replicates containing six rabbits. After an adaptation period of 7 d during which the rabbits were fed, *ad libitum*, a transition from standard to experimental diet was followed by a 40 d feeding trial. Natural lighting and automatic ventilation were used in the rabbit house; the temperature ranged from 12°C to 24°C, and the relative humidity ranged from 40% to 50% throughout the trial. Determination of digestive energy by the endogenous indicator method was conducted in the last week of the feeding trial.

### Diets and experimental procedures

The diets corresponded to a linear decrease in starch level (28% to 16%) and to a nearly linear increase in dF (16% to 23%); dF/starch ratios were 0.59, 0.66, 0.71, 1.05, and 1.44. The diets were approximately isoenergetic and isonitrogenous. Table 1 shows the diet composition and nutrient levels that were determined following rabbit nutrient demand suggested by Lebas et al [13], as well as considerations of local feed sources in Shenyang, China. Each treatment group was fed with one of the five different diets. The feed was pressed using a flat-mode pellet mill and a multi-function counter-flow dryer (KL-260, Laizhou, China) into pellets approximately 4 mm in diameter and 10 mm long.

### Chemical analyses of the diets

The dietary gross energy was analyzed by a full-automatic calorimeter (IKA C2000, Staufen, Germany). Crude protein was analyzed by a semi-automated Kjeldahl technique. Neutral detergent fiber (NDF), ADF, and acid detergent lignin used detergent method of Van-Soest by an automatic fiber analyzer (ANKOM A2000i, Macedon NY, US). Starch was measured according to national food safety standard (GB 5009.9-2016 Determination of starch in foods).

### Determination of the pellet quality

#### Particle size

We used a 150 mm long vernier caliper, with a precision of 0.02 mm, to determine particle size.

#### Bulk density (accumulation density, kg/L)

We filled the feed pellets into a 1,000 mL cylinder, leveled the particles off on the top edge of the cylinder, weighed this sample, and calculated the weight to volume ratio.

#### Hardness

We used a sclerometer (ST120A, Jinan, China) to determine pellet hardness.

#### Powder ratio

Powder ratio determination was based on the national standard (GB/T 16765-1997). We sieved the sample with a 2-mm metal sieve, weighed the particles passing through the screen, and calculated the percentage of the weight of screened particles to the total weight of the sample.

#### Pellet durability index

We used the national standard (GB/T 5169-91) and a pellet durability index (PDI) instrument (ST-136, Jinan, China) to score the PDI.

The number of replications for particle size, bulk density, hardness, powder ratio, and PDI determination of feed pellet were 30, 5, 15, 3, and 3 respectively.

### Performance determination

#### Growth performance

At the start and end of the experiment, rabbits were weighed in the morning. The feed consumed was recorded daily. Weight gain, feed/gain (F/G), and wool yield were measured after the feeding trial.

#### Wool yield

All of the hair shed by each rabbit was individually weighed.

#### Fiber length

Fiber samples were collected from a 10×10 cm patch area in the rib area of the left side of each rabbit, and their lengths were measured using a ruler. A total of 200 fibers from each rabbit were measured.

#### Fiber diameter

Diameters of fiber samples were measured using a fiber fineness meter (CU-2, Guoliangyiqi Inc., Wuhan, China).

### Sampling and analysis of cecal microbiota

#### Slaughtering and sampling

At 123 d of age (at the shearing period), 20 rabbits (4 per group) in apparently healthy condition were euthanized by sudden cervical dislocation. For each rabbit, the cecal digesta was collected in tube and stored at −80°C until processed.

#### Genomic DNA extraction

Genomic DNA from the cecal digesta (0.2 g) was extracted using the cetyltrimethylammonium bromide (CTAB) method. The extracted DNA (2 μL) was then subjected to electrophoresis on a 7 g/L agarose gel (W/V) to estimate the amount and integrity of the DNA products. The DNA solution was preserved at −20°C until further analysis.

#### Polymerase chain reaction amplification

Amplification of the variable V3-V4 region of bacterial 16S rDNA was performed using the general primer sequences 340F and 805R. The details of these primers are as follows: upstream primer 340F: 5′-CCTACGGGNBGCASCAG -3′; downstream primer 805R: 5′-GACTACNVGGGTATCTAATCC -3′. Polymerase chain reaction (PCR) was performed using model PTC220 (Bio-Rad Laboratories Inc., Hercules, CA, USA) and PCR amplification kits (KAPA Biosystems, Cape Town, South Africa) in reaction mixtures containing 5 μL 10× PCR buffer; 1 μL dNTP (2.5 mM); 0.2 μL rTaq (5 U/μL); 2 μL 340F (20 mM); 2 μL 805R (20 mM); 1 μL template DNA, and made up to a final volume of 50 μL using double distilled water. The thermal cycle used the following conditions: initial denaturation at 95°C for 3 min, followed by 30 cycles at 95°C for 30 s, 58°C for 30 s, and 72°C for 60 s, and final elongation at 72°C for 7 min.

#### Mixing and purification of polymerase chain reaction products

Samples were mixed according to the concentration of the PCR products. After complete mixing, the PCR products were purified using 0.5×tris-boric acid buffer of 1.5% agarose gel electrophoresis. The targets were then recovered using a MinElute Gel Extraction Kit (QIAGEN Biotechnology Co., Ltd., Shanghai, China).

#### Library construction and sequencing

The complementary DNA Library was constructed using a NEB Next Ultra DNA Library Prep Kit (New England Biolabs, Ltd., Beijing, China) for Illumina. The constructed library was tested by Qubit quantitative and library and, after qualification, MiSeq 2500 was used for sequencing.

#### Bioinformatics analysis

Paired-end reads assemblies: We used FastQC software to evaluate the number and quality of sequencing. Paired-end reads from the original DNA fragments were merged using FLASH v1.2.7 http://ccb.jhu.edu/software/FLASH/. Paired-end reads were assigned to each sample according to the unique barcodes and follow up analysis with the processed sequence; operational taxonomic units (OTUs) cluster and Species annotation. Sequence analysis was performed by QIIME 1.8 software package [14]. We filtered out a single read base in which the number of bases with scores above 30 points accounted for less than 80% of the reads using the command split_libraries_fastq.py in QIIME1.8. We removed chimera by USEARCH v6.1, and followed up the analysis using filtered sequence. Statistics of reads for each sample and cluster analysis was completed according to sequence similarity using uclust v1.2.22. OTUs used 97% as a similarity threshold. Follow up analyses of species and diversity indexes were completed using the standardized OTU file. The standardized threshold was 200,000 sequences. We used ribosomal database project classifier software and the green genes database for species annotation, and statistics of the species composition for each sample at the phylum, family and genus levels. A community map was drawn of multi-sample species composition. To compute Alpha diversity, we rarified the OTU table and calculated three metrics: The Chao1, the Observed species, and the Shannon index.

### Statistical analysis

Data were analyzed using one-way analysis of variance with SPSS 21.0 (SPSS Inc., Chicago, IL, USA). Contrast was established using linear and quadratic polynomial methods to determine the effects of increasing the dF/starch ratio. The data are presented as means, standard error of the mean, and p-values. Multiple comparisons were made using least significant difference method. Differences were considered significant at p<0.05.

## RESULTS

### Pellet quality

Effects of the dietary dF/starch ratio on pellet quality are presented in Table 2. The dietary dF/starch ratio had a significant effect on all of the variables (p<0.05) except for pellet diameter. Additionally, all of the pellets variables had linear responses (p<0.01). Significant quadratic effects (p<0.01) on bulk density (y_1_, p<0.001) and hardness (y_2_, p<0.05) were observed. Based on the regression equation (y_1_ = −0.11x^2^+0.14x+0.53, R^2^ = 0.78; y_2_ = −10.43x^2^+31.83x−9.84, R^2^ = 0.58), when the dF/starch ratio was 0.67, the pellet bulk density was the highest, and when the dF/starch was 1.53, the hardness was the greatest. When the dF/starch ratio was 1.44, the feed had the best pellet quality (the lowest powder ratio and the highest PDI).

### Production performance

Effects of different dietary dF/starch ratios on growth and wool yield in Angora rabbits are shown in Table 3. The rabbits were healthy and appeared to be in normal condition during the experimental period. The average daily gain (ADG) in the 1.44 dF/starch treatment was the lowest among all treatments (p<0.05). The ADG in the 0.59 and 0.71 dF/starch treatments were significantly higher than that in the 1.05 dF/starch treatment (p<0.05), The average daily feed intake (ADFI) in the 0.71 dF/starch treatment was higher than that in the 0.59 and 0.66 dF/starch treatments (p<0.05), and these were higher than the ADFI in the 1.05 and 1.44 dF/starch treatments (p< 0.05). The F/G in the 1.44 dF/starch treatment was the highest among all treatments (p<0.05). Mortality was not affected by any of the dietary treatments (p>0.05) and there was no obvious change rule. There were significantly linear reduction effects on ADG and ADFI with the increase of the dietary dF/starch ratio (p<0.001). Quadratic effects on ADG and F/G (p<0.05) were also observed. Based on the regression equations (Table 4), when the dF/starch ratio was 0.59, the ADG was the highest, and when the dF/starch ratio was 0.74, the F/G was the lowest.

The wool yield in the 0.66 dF/starch treatment was the highest (140.6 g), and this was higher than that in the 1.44 dF/starch treatment by 9.37% (p<0.05), but there was no significant difference compared with the other groups (p>0.05). The fiber diameter in the 1.05 dF/starch treatment was the finest, and this was finer than that in the 0.59 dF/starch treatment (p<0.05).The shortest fiber length (5.63 cm) occurred in the 0.66 dF/starch treatment and this was shorter than that in the 1.05 dF/starch treatment by 17.81% (p<0.05). A significant favorable quadratic effect on fiber diameter was seen (p<0.05). Based on the regression equation (Table 4), when the dF/starch ratio was 1.06, the fiber diameter was the finest.

### Cecal microbiota

The microbial 16S rDNA V3-V4 region of 20 cecum contents in 5 treatments were sequenced based on Illumina Miseq sequencing technology, in which 18 samples met the requirements. OTUs were classified according to the similarity of 97%; the effective sequence range of each sample was 239,496 to 963,776 and the range of OTUs was 4,403 to 10,959.

#### Cecal microbial alpha diversity

Alpha diversity is the analysis of species diversity within a single sample. Table 5 shows that the Good’s coverage was above 0.98. The sequencing therefore had high coverage for bacteria and was suitable for the analysis of bacterial diversity. The OTU number index in the 1.05 dF/starch treatment was significantly higher than that in the 0.66 and 0.71 dF/starch ratio treatments. As the dF/starch ratio increased, the cecal microbiota OTU number index increased (p<0.05) but no effects on cecal microbiota Abundance, Simpson and Shannon index, or Good’s coverage were observed (p>0.05). However, there was a significant relationship between the sequencing coverage (p<0.05), which decreased linearly with an increase of dF/starch. This indicated that most of the bacteria were covered, but a small number of bacteria remained undetected.

#### Microbial species annotation

**Figure 1:**
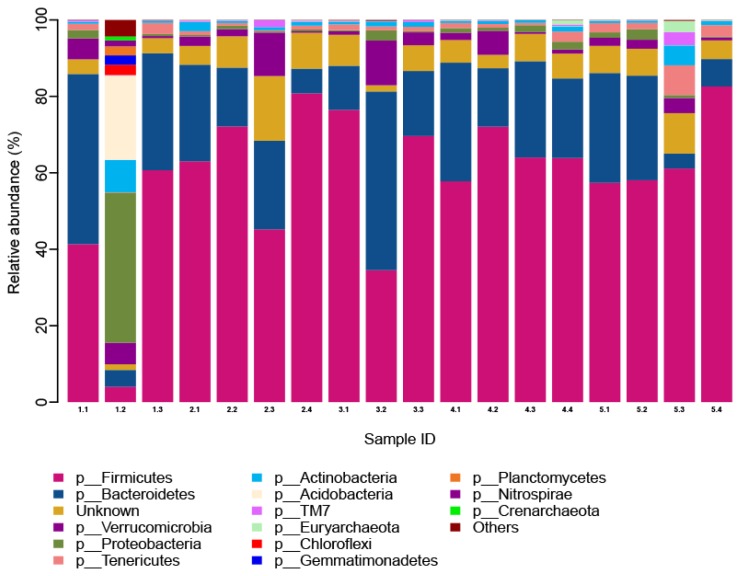
Analysis of cecal microbial community structure in the Angora rabbits (at phylum level). Cecal microbiota was detected by using the second generation high-throughput sequencing platform based on llumina MiSeq 2500. We used ribosomal database project classifier software and the greengenes database for species annotation. There were 12 phyla with proportions greater than 0.1%. These were: Firmicutes (59.14%), Bacteroidetes (21.38%), Verrucomicrobia (3.47%), and et al. The horizontal axis is the identifier of each sample (Lanes 1.1, 1.2, 1.3; 2.1, 2.2, 2.3, 2.4; 3.1, 3.2, 3.3; 4.1, 4.2, 4.3, 4.4, and 5.1, 5.2, 5.3, 5.4 stand for the 0.59, 0.66, 0.71, 1.05, and 1.44 dF/starch ratio groups, respectively), the longitudinal axis is relative abundance. The color corresponds to the name of each species at phylum level, and the relative abundance ratio of different species is represented by different color block widths.

We annotated the microbial species found in this study. A total of 14 phyla, 24 classes, 26 orders, 25 families, and 17 genera were obtained from the 18 samples. The effect of different dF/starch ratios on the proportion of microbiota phylum is shown in Figure 1. There were 12 phyla with proportions greater than 0.1%. These were: Firmicutes 59.14%, Bacteroidetes 21.38%, Verrucomicrobia 3.47%, Proteobacteria 3.25%, Tenericutes 1.77%, Actinobacteria 1.57%, Acidobacteria 1.26%, TM7 0.44%, Euryarchaeota 0.29%, Chloroflexi 0.16%, Gemmatimonadetes 0.14%, and Planctomycetes 0.13%.

The cecum microbiota was similar at the phylum level, among the different dietary dF/starch ratio treatments (p> 0.05). The dominant bacteria phyla were Firmicutes and Bacteroidetes, followed by Verrucomicrobia and Proteobacteria. The relative microbiota abundance of Bacteroidetes, Proteobacteria, Actinobacteria, Acidobacteria, Chloroflexi, Gemmatimonadetes, Planctomycetes, as well as Nitrospirae, and Crenarchaeot in the 0.59 dF/starch treatment was the highest among all other groups. The microbiota abundance of Firmicutes in the 0.66 dF/starch treatment was the highest among all other treatments. The relative microbiota abundance of Verrucomicrobia in the 0.71 dF/starch treatment was the highest among all treatments. The 1.44 dF/starch treatment had the largest proportion of Tenericutes, TM7 and Euryarchaeota.

At the genus level, 14 shared bacteria taxa were identified (Table 6). The percentage of unknown bacteria was 77.18%. Shared bacteria with percentages >1.0% were: *Ruminococcus* (4.58%), *Bacteroides* (4.88%), *Akkermansia* (3.21%), *Faecalibacterium* (2.43%), and *Oscillospira* (1.22%). The abundance ratio of bacteria at the genus level was not affected by the dF/starch ratio (p>0.05) except for *Ruminococcus*. The proportion of *Ruminococcus* in the 0.71 dF/starch treatment was higher than that in the 0.59 dF/starch treatment (p<0.05).There were 3 genera (*DA101*, *Candidatus Solibacter* and H*yphomicrobium*) found only in the 0.59 dF/starch treatment.

#### Operational taxonomic units comparison of the microbiota (heatmap)

**Figure 2:**
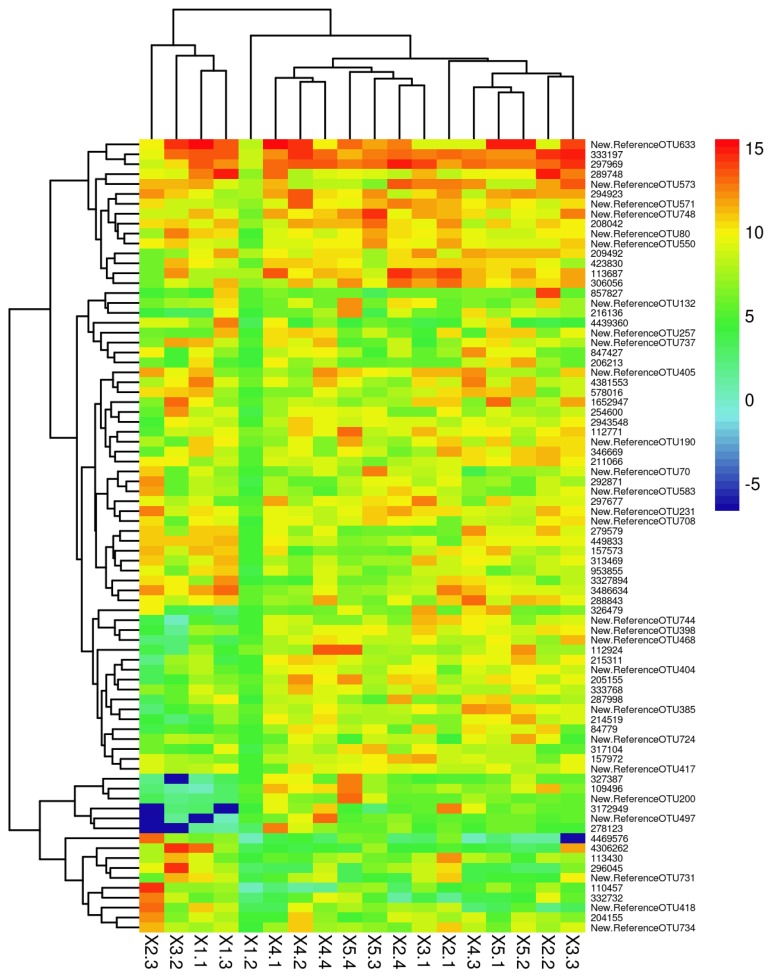
Operational taxonomic units (OTUs) heatmap of cecal microbial constitution in Angora rabbits. The heamap of species abundance is drawn by using gplots package of R software. Each column represents a sample, each row represents an OTU and species information corresponding to the OTU, color block represents species abundance relative values, and the redder the color, the higher the relative abundance, the blue the opposite. The ruler on the right is the color key and value. The left side of the graph shows the microbiota clustering. In addition, the heatmap clustered the samples. The more similar the distribution of sample microflora, the closer the sample distance is, and the closer the location is in the cluster tree above the graph. Column X2.3, 3.2, 1.1 and 1.3 grouped into a cluster, the remaining treatments grouped into a separate cluster. X1.1, 1.2, 1.3; X2.1, 2.2, 2.3, 2.4; X3.1, 3.2, 3.3; X4.1, 4.2, 4.3, 4.4 and X5.1, 5.2, 5.3, 5.4 were samples from the 0.59, 0.66, 0.71, 1.05, and 1.44 dF/starch ratio groups, respectively. The heatmap represents the top 80 OTUs. Each OTU corresponding species information refers to the greengenes database (http://greengenes.secondgenome.com/).

To reflect OTU positioning among the samples, two dimensional clustering of samples and OTUs was conducted, resulting in a graphical heatmap (Figure 2). In the heatmap, each column represents a sample and each row (small grid) represents an OTU and the species information corresponding to the OTU. The color represents the number of reads contained in the OTU (red, high; blue, low). The similarity and differences of multiple sample communities were reflected by the color gradient and similarity. Two clusters are shown in Figure 2. The left cluster includes column X2.3, 3.2, 1.1 and 1.3 that originated from the 0.66, 0.71, 0.59, and 0.59 dF/starch treatments, respectively; the remaining treatments grouped into a separate cluster. The color of the left cluster was lighter than that of the right cluster. This indicates that the greater the dF/starch ratio, the higher the bacterial abundance.

At the family level, unknown bacteria accounted for 33.46% of the total, followed by *Ruminococcaceae* (26.13%), S247 (10.42%), *Lachnospiraceae* (10.39%), *Bacteroidaceae* (4.63%), *Verrucomicrobiaceae* (3.16%), and *Rikenellaceae* (2.90%). Other families (*Coriobacteriaceae*, *Porphyromonadaceae*, *Barnesiellaceae* accounted for less than 1%. The proportion of bacterial populations among treatments was similar except for the *Lachnospiraceae*. The proportion of cecal *Lachnospiraceae* in the 0.66 dF/starch treatment was significantly higher than in other treatments (p<0.05).

## DISCUSSION

### Effects of digestible fiber-to-starch ratio on pellet quality in Angora rabbits

The pellet quality of rabbits is mainly a function of the particle size, pellet durability and hardness. The diameter and length of the pellets affect the feeding, digestion and processing cost of rabbits. The length of pellets is preferentially between 8 to 10 mm [15]. If pellets are longer than 8 to 10 mm, there is a higher risk of breakage during handling. The optimal pellet diameter ranges from 3 to 4 mm. This diameter is suitable for use in rabbit feeders and minimizes production costs. At pellet diameters >5 mm, pellet wastage increases [2]. In this study, we observed that the dietary dF/starch ratio had a direct correlation with pellet length (7.49 to 10.9 mm) and diameter (3.94 to 4.09 mm). Therefore, the size of the pellets tested was within the optimal range.

Pellet durability and hardness are major quality charac teristics of rabbit pellets because rabbits do not eat the fines between the pellets. When provided diets with a mixture of pellets of different hardness, rabbits tend to eat a greater percentage of the hard particles. However, very hard pellets with low elasticity are fragile and can produce a large amount of fines [2]. Morisse et al [16] reported that when the resistance to crushing was between 7 and 13 kg, the biological performance of rabbits was not influenced by pellet hardness. We observed that dietary dF/starch had significant linear increasing effects on the PDI (91.6% to 96.6%) and hardness (5.82 to 14.11 kg), but had linear decreasing effects on bulk density and the powder ratio. This was consistent with the findings of Yang et al [17], who demonstrated that higher fiber (16% crude fiber) was associated with greater pellet hardness (9.57 kg). Acedo-rico et al [2] showed that ingredients with high cellulose levels are more flexible in processing and produced better pellet quality. This is due to the polymerization of cellulose. Yu et al [18] reported that addition of cellulose can increase pellet hardness. However, when the cellulose in the formula is excessive, the pellets are not easily extruded. Therefore, based on the special digestive physiology of rabbits, the preparation of a moderately high dF/starch diet has beneficial effects on pellet quality. In this study, the 1.44 dF/starch ratio was optimal.

### Effects of digestible fiber-to-starch ratio on growth of Angora rabbits

The digestive energy present in rabbit diet is mainly from starch and dF but excessive dF will reduce rabbit growth. Perez et al [4] reported that the replacement of 10 percentage points of starch by dF reduced the rabbit diarrhea rate without altering rabbit growth or feeding efficiency. De Blas et al [11] reported that the optimal values for dietary starch and NDF contents in rabbit doe diets were approximately 20% and 35.5% (as dry matter basis). In the present study, when the dF/starch ratio increased, the rabbit growth properties significantly decreased, but without changing the mortality rate, wool yield, or fiber diameter. Trocino et al [19] also reported that neither the dietary soluble fiber to starch ratio nor the protein level affected the health status of growing rabbits.

We found that a diet combining a low dF level with rela tively high starch (dF/starch: 0.59 to 0.74) enhanced rabbit growth. These results are consistent with the results of Tazzoli et al [20] who observed that final live weight and ADG significantly decreased with decreasing starch from 19.6% to 11.5% in rabbit diets, El-Tahan et al [21] reported that feeding a high starch diet (up to 22.11%) provided the best growth performance and digestibility coefficients in growing meat rabbits. The impairment of rabbit growth caused by feed restriction was enhanced when the dF/starch ratio was increased [22]. Although Xiccato et al [23] indicated that the dF/starch ratio (1.0 vs 2.5) did not affect the final live weight and ADG of hybrid rabbits. Therefore, if only the growth performance is considered, the optimal dF/starch ratio for Angora rabbits in this study was 0.59 to 0.74. Zhou et al [24] demonstrated that the best dF/starch ratio for production of meat rabbits was 0.99 (dF 19.89%, starch 20.11%).

For regular diets, without lipid addition, growing rabbits regulate their feed intake based on the digestible energy level of the diet [3]. If the digestible energy levels are equivalent between the diets the intake levels would be expected to be similar. Surprisingly, a higher correlation of the ADFI with the dF/starch ratio was found in this study. However, previous study has also found a decreased ADFI with the use of diets rich in dF [20]. This result is related to the lower bulk density of high dF/starch diets.

In this paper, the highest wool yield was obtained in the 0.66 dF/starch treatment, but there was no significant difference compared with the other groups except for the 1.44 dF/starch ratio treatment. Some impairment in the performances of Angora rabbits was observed in those fed the highest dF/starch ratio (1.44). This might be explained by higher fermentation losses in the cecum [11]. The estimated optimal ratio for fiber diameter by the quadratic equation was 1.06. No specific study has been published on dietary dF/starch ratio as a possible source of variation in Angora wool growth. Owing to one of the roles of dietary fiber is to remove hair swallowed by the rabbit from the digestive tract [13], as well as considering the wool yield, fiber diameter, and feed cost, a digestible dF/starch ratio of 1.06 is suggested for wool performance.

### Effects of digestible fiber-to-starch ratio on the cecal microbiota in Angora rabbits

Fiber degradation is ultimately determined by microbial activity, digesta retention time in the cecum and fiber chemical composition and structure [25]. Jehl and Gidenne [26] showed that replacement of starch by dF appeared to favor fermentative and microbial activity without affecting the ceco-rectal rate of passage. Greater enzymatic activity involved in degrading pectin and hemicelluloses than for degrading cellulose has been documented [25]. These results correspond to the fecal digestibility of the corresponding dietary fiber constituents in rabbits, and are consistent with the lower counts of cellulolytic bacteria in the rabbit cecum [27].

We found that the cecal microbiota remained relatively stable at the phylum level. The dominant bacteria phyla (%) were Firmicutes (59.14), Bacteroidetes (21.38), Verrucomicrobia (3.47), and Proteobacteria (3.25). This result was consistent with results of Xu et al [28] who reported that the cecal microbiota of healthy young rabbits at different ages mainly consisted of Firmicutes (39.18%), Bacteroidetes (22.66%), Proteobacteria (8.75%), and Verrucomicrobia (1.84%). Other researchers have reported similar results [29].

*Ruminococcus* spp. is the main Fibrolytic bacteria found in the rumen of cattle [30]. We demonstrated that the abundance ratio of *Ruminococcus* in the rabbit cecum in all treatments exceeded 1%, followed by *Bacteroides* and *Akkermansia*. These data could explain why both high dF and high starch diets can promote the growth of *Fibrolytic bacteria*. However, when the dF/starch ratio was 0.71, the abundance ratio of *Ruminococcus* was the highest (6.24%). So, the 0.71 dF/starch diet seems to be more beneficial to the decomposition of fiber and this result was consistent with the growth rate. We speculate that appropriate dF/starch ratio diet can provides both long and digestible fiber, which allows an adequate transit time of the digesta and a balanced growth of the cecal microbiota. There was a high percentage (77.18%) of unidentified bacteria genera in this study, indicating that there are many unidentifiable species of intestinal microbiota in Angora rabbits. More attention should be given to the characteristics and functions of unknown bacteria for a better understanding of the intestinal microbiota.

## CONCLUSION

In conclusion, the most suitable dF/starch ratio for feed pellet quality is 1.44 and for rabbit growth the optimal range of ratios is from 0.59 to 0.74. In addition, for wool growth, the optimal range of ratios is from 0.66 to 1.06. With the combination of the output cost and cecal microbiota, we suggest that a dietary dF/starch ratio for Angora rabbits ranging from 0.74 to 1.06 is optimal.

## Figures and Tables

**Table 1:** Ingredients and chemical composition of the experimental diets

Items	Digestible fibre1)-to-starch ratio				

	0.59	0.66	0.71	1.05	1.44
Ingredient (%, as fed)					
Maize grain	36.22	33.60	29.40	23.00	14.60
Soya bean meal	23.00	19.65	18.20	14.70	12.30
Wheat bran	3.60	11.40	20.00	20.00	20.00
Rice bran	3.00	3.00	3.00	3.00	3.00
Grass meal	3.50	3.50	0.60	22.60	38.60
Rice husk	24.18	22.35	22.30	10.20	5.00
A mixture of rice flour, germ and rice bran	4.00	4.00	4.00	4.00	4.00
Premix2)	2.50	2.50	2.50	2.50	2.50
Nutrition composition (%, as dry matter)3)					
Dry matter	86.71	86.62	86.53	86.72	86.91
Digestive energy (MJ/kg)	10.82	11.04	10.74	10.68	10.81
Crude protein	16.40	16.22	16.28	16.16	16.43
NDF	34.36	33.54	35.67	40.48	36.87
ADF	20.80	18.11	19.63	20.80	17.66
ADL	5.21	5.11	5.24	4.11	3.91
Water insoluble pectin	2.82	2.76	2.74	3.29	3.74
Starch	27.96	27.70	26.44	21.89	15.98
Hemicelluloses (NDF-ADF)	13.56	15.44	16.04	19.68	19.21
Digestible fibre1)	16.38	18.20	18.78	22.98	22.95
Ca	0.83	0.82	0.81	0.91	0.91
P	0.60	0.65	0.72	0.75	0.76

NDF, neutral detergent fiber; ADF, acid detergent fiber; ADL, acid detergent lignin.

1) Digestible fibre was calculated by hemicelluloses + water insoluble pectin.

2) Supplied the following per kg of diet: vitamin premix 0.2 g; mineral premix 1.0 g; salt 3 g, *_L_*-Lys·H_2_SO_4_ 3.3 g; *_DL_*-Met 4.0 g; mycotoxin adsorbent 0.3 g; antioxidant 0.2 g; limestone 13 g.

3) Nutrient levels were measured values except water insoluble pectin, which were calculated according to tables of ingredients [12].

**Table 2:** Effect of the dietary digestible fibre-to-starch ratio on feed pellet quality of Angora rabbits

Parameters	Digestible fibre1)-to-starch ratio					SEM	p-values	
	
	0.59	0.66	0.71	1.05	1.44		Linear	Quadratic
Bulk density (g/mL)	0.56^b^	0.58^a^	0.58^a^	0.55^b^	0.51^c^	0.01	<0.001	<0.001
Pellet length (mm)	9.36^b^	10.07^ab^	7.49^c^	9.79^ab^	10.90^a^	0.22	0.001	0.132
Pellet diameter (mm)	3.9^4^	4.03	3.97	4.04	4.09	0.21	0.009	0.848
Powder ratio (%)	5.49^a^	3.48^ab^	3.94^ab^	2.60^b^	1.48^b^	0.47	0.007	0.469
Durability index (%)	91.79^bc^	91.57^c^	92.52^bc^	94.58^ab^	96.63^a^	0.61	0.001	0.842
Hardness (kg)	6.77^b^	6.12^b^	5.82^b^	13.05^a^	14.11^a^	0.53	<0.001	<0.036

SEM, standard error of the mean.

1) Digestible fibre was calculated by hemicelluloses + water insoluble pectin.

a–c Values within a row with different superscripts differ significantly at p<0.05.

**Table 3:** Effects of the dietary digestible fibre-to-starch ratio on growth and wool yield in Angora rabbits

Performance	Digestible fibre1)-to-starch ratio					SEM	p-values	
	
	0.59	0.66	0.71	1.05	1.44		Linear	Quadratic
Initial weight (g)	2,192	2,222	2,255	2,173	2,125	16.5	0.097	0.041
Average daily gain (g)	20.37^a^	19.21^ab^	20.06^a^	18.01^b^	12.95^c^	0.67	<0.001	0.033
Average daily feed intake (g)	141.75^b^	144.45^b^	149.6^a^	137.71^c^	136.8^c^	1.14	<0.001	0.929
Feed/gain	6.98^b^	7.53^b^	7.50^b^	7.65^b^	10.75^a^	0.34	<0.001	0.010
Mortality (%)	4.17	16.67	12.50	8.33	12.50	2.78	0.853	0.988
Wool yield (g)	132.53^ab^	140.6^a^	138.13^ab^	133.03^ab^	127.43^b^	2.32	0.150	0.576
Fiber length (cm)	6.55^a^	5.63^b^	6.60^a^	6.85^a^	6.00^ab^	0.15	0.800	0.072
Fiber diameter (μm)	16.62^a^	15.38^ab^	15.05^ab^	14.61^b^	15.47^ab^	0.26	0.333	0.040

SEM, standard error of the mean.

1) Digestible fibre was calculated by hemicelluloses + water insoluble pectin.

a–c Values within a row with different superscripts differ significantly at p<0.05.

**Table 4:** The quadratic regression equations, coefficients of determination, and extremums of the Angora rabbits

Parameter	Quadratic regression equation	R^2^	Extremum1),2)
ADG	−9.63x^2^+11.32x+16.64	0.85	0.59
F/G	7.21x^2^−10.73x+11.19	0.79	0.74
Wool fiber diameter	8.20x^2^−17.40x+23.59	0.80	1.06

R^2^, coefficient of determination; ADG, average daily gain; F/G, feed/gain.

1) The extremum is expressed as the dietary digestible fiber-to-starch ratio.

2) The digestible fiber-to-starch ratio estimates represent of the maximum or minimum response.

**Table 5:** Effect of the dietary digestible fibre-to-starch ratio on cecal microbial alpha diversity in Angora rabbits

Items	Digestible fibre1)-to-starch ratio					SEM	p-values	
	
	0.59	0.66	0.71	1.05	1.44		Linear	Quadratic
OTU number index (Chao 1 value)	11,313abc	9,673^c^	9,877^bc^	12,567^a^	11,208^ab^	443.4	0.025	0.551
OTU number index (ACE value)	11,838	10,427	10,282	13,357	13,292	505.4	0.052	0.251
Simpson index	0.969	0.967	0.966	0.966	0.973	0.004	0.606	0.617
Shannon index	8.21	6.90	6.88	7.36	7.60	0.243	0.846	0.302
Good’s coverage	0.988	0.988	0.988	0.984	0.984	<0.001	0.019	0.696

SEM, standard error of the mean; OTU, operational taxonomic unit; Chao 1, the Chao 1 estimator; (http://www.mothur.org/wiki/Chao); ACE, the ACE estimator (http://www.mothur.org/wiki/Ace).

1) Digestible fibre was calculated by hemicelluloses + water insoluble pectin.

a–c Values within a row with different superscripts differ significantly at p<0.05.

**Table 6:** Effect of the different dietary digestible fibre-to-starch ratio on proportion of microbiota genus in cecal digesta of the Angora rabbits (%)

Microbiota genus	Digestible fibre1)-to-starch ratio					SEM	p-values	
	
	0.59	0.66	0.71	1.05	1.44		Linear	Quadratic
Unknown	77.26	75.8	72.44	79.93	80.49	1.74	0.255	0.927
*Ruminococcus*	1.88^b^	5.23^ab^	6.24^a^	4.83^ab^	4.68^ab^	0.57	0.686	0.302
*Bacteroides*	6.93	5.33	7.43	3.37	1.34	1.08	0.071	0.937
*Akkermansia*	2.04	4.01	5.3	2.41	2.29	0.83	0.529	0.866
*Faecalibacterium*	0.71	1.53	2.75	2.38	4.78	0.64	0.065	0.815
*Oscillospira*	0.60	1.58	1.12	1.54	1.28	0.17	0.551	0.278
*Parabacteroides*	0.85	1.13	1.33	0.74	0.82	0.24	0.661	0.934
*Clostridium*	0.25	2.09	0.70	0.24	0.27	0.32	0.245	0.817
*Desulfovibrio*	0.56	0.28	0.66	0.91	0.78	0.15	0.353	0.528
*Odoribacter*	0.54	0.58	0.28	0.94	0.35	0.14	0.957	0.272
*Rikenella*	0.46	0.23	0.15	0.73	0.54	0.11	0.264	0.492
*Coprococcus*	0.20	0.31	0.10	0.67	0.53	0.12	0.258	0.453
*Methanobrevibacter*	0.01	0.09	0.07	0.33	0.80	0.17	0.110	0.812
*Rhodoplanes*	1.59	0.01	0.01	0.01	0.01	0.26	0.313	0.312
*Blautia*	0.17	0.62	0.18	0.10	0.20	0.08	0.332	0.509
*DA101*	0.88	0	0.01	0.01	0	0.15	0.313	0.312
*Candidatus Solibacter*	0.78	0	0	0.01	0.01	0.13	0.316	0.312
*Hyphomicrobium*	0.41	0	0	0	0	0.07	0.312	0.311
Others	3.88	1.19	1.24	0.84	0.83	0.43	0.136	0.217

SEM, standard error of the mean.

1) Digestible fibre was calculated by hemicelluloses + water insoluble pectin.
